# Fragile X syndrome screening in Chinese children with unknown intellectual developmental disorder

**DOI:** 10.1186/s12887-015-0394-8

**Published:** 2015-07-15

**Authors:** Xiaoli Chen, Jingmin Wang, Hua Xie, Wenjuan Zhou, Ye Wu, Jun Wang, Jian Qin, Jin Guo, Qiang Gu, Xiaozhen Zhang, Taoyun Ji, Yu Zhang, Zhiming Xiong, Liwen Wang, Xiru Wu, Gary J. Latham, Yuwu Jiang

**Affiliations:** Municipal Key Laboratory of Child Development and Nutriomics, Capital Institute of Pediatrics, Beijing, China; Department of Pediatrics, Peking University First Hospital, Beijing, China; Department of Neurology, Affiliated Children’s Hospital of Capital Institute of Pediatrics, Beijing, China; Beijing Microread Genetech Co., Ltd, Beijing, China; Department of Genetics, Jiangxi Previncial Children’s Hospital, Jiangxi, China; State Key Lab of Medical Genetics, Central South University, Changsha, China; Research & Technology Development, Asuragen, Inc., Austin, TX USA

**Keywords:** Chinese children, Intellectual developmental disorder, *FMR1*, Fragile X syndrome, Triplet repeat primed (TP)-PCR

## Abstract

**Background:**

Fragile X syndrome is the most common genetic disorder of intellectual developmental disorder/mental retardation (IDD/MR). The prevalence of FXS in a Chinese IDD children seeking diagnosis/treatment in mainland China is unknown.

**Methods:**

Patients with unknown moderate to severe IDD were recruited from two children’s hospitals. Informed consent was obtained from the children's parents. The size of the CGG repeat was identified using a commercial TP-PCR assay. The influence of AGG interruptions on the CGG expansion during maternal transmission was analyzed in 24 mother-son pairs (10 pairs with 1 AGG and 14 pairs with 2 AGGs).

**Results:**

553 unrelated patients between six months and eighteen years of age were recruited. Specimens from 540 patients (male:female = 5.2:1) produced high-quality TP-PCR data, resulting in the determination of the *FMR1* CGG repeat number for each. The most common repeat numbers were 29 and 30, and the most frequent interruption pattern was 2 or 3 AGGs. Five full mutations were identified (1 familial and 4 sporadic IDD patients), and size mosaicism was apparent in 4 of these FXS patients (4/5 = 80 %). The overall yield of FXS in the IDD cohort was 0.93 % (5/540). Neither the mean size of CGG expansion (0.20 vs. 0.79, *p* > 0.05) nor the frequency of CGG expansion (2/10 vs. 9/14, *p* > 0.05) was significantly different between the 1 and 2 AGG groups following maternal transmission.

**Conclusions:**

The *FMR1* TP-PCR assay generates reliable and sensitive results across a large number of patient specimens, and is suitable for clinical genetic diagnosis. Using this assay, the prevalence of FXS was 0.93 % in Chinese children with unknown IDD.

**Electronic supplementary material:**

The online version of this article (doi:10.1186/s12887-015-0394-8) contains supplementary material, which is available to authorized users.

## Background

Intellectual developmental disorder/mental retardation (IDD/MR) encompasses a cluster of symptoms that are characterized by low intelligence and limitations in adaptive behavior and functional capabilities [1, 2]. Generally, IDD occurs in approximately 1-3 % of individuals worldwide, with an incidence of 1 % in high income countries and 2 % in low/middle income countries [3, 4].

Genetic/genomic factors are a major risk factor for IDD, accounting for 85 % of patients with IDD [5]. Among them, fragile X syndrome (FXS, MIM 309550) is the most common form of IDD. The prevalence of FXS is estimated to be 1/4,000 in males and 1/5,000-8000 in females [6, 7]. FXS accounts for approximately 20 % of patients with X-linked IDD [8] and 2–7 % of children with autism [9, 10]. However, the classic facial features of FXS (prominent forehead, a long narrow face, protruding ears, and macroorchidism) are ambiguous until juvenile development, and neurophysical symptoms are also subtle in young children [11]. The lack of a clear phenotype in young children can delay a definitive diagnosis, leading to a diagnostic “odyssey” for families and a delay in the implementation of specific therapies. Indeed, it has been reported that in the USA up to 40 % of families with FXS girls and 25 % with FXS boys have had another child before their first affected child was diagnosed [12]. Additionally, only a 40 % penetrance for mental impairment is reported in affected females [13, 14], making a clear clinical diagnosis even more difficult in girls. Consequently, identifying children, particularly infants and toddlers with FXS, is critically dependent on molecular genetics testing.

FXS is typically caused by an expansion of CGG trinucleotides repeats in the 5′ untranslated region of the *FMR1* gene. The most common size of CGG repeats among the general population is 29 and 30 copies [15]. In FXS patients, the CGG number expands to greater than 200 repeats, resulting epigenetic silencing of the *FMR1* gene and the absence of the encoded protein, fragile X mental retardation protein (FMRP) [15]. FXS molecular diagnostic tests include region-specific CGG PCR amplification and Southern blot (SB) analysis. Recently, triplet repeat-primed (TP)-PCR methods have been described, which simplify *FMR1* genotyping and can detect both full mutation expansions and low-level size mosaicism with high sensitivity [16, 17].

The prevalence of FXS in a Chinese IDD population has been previously reported [18, 19] However, the rate of FXS in IDD children seeking diagnosis/treatment in mainland China is unknown. The availability of such information is expected to help enhance awareness among neurologists in suspicious populations and improve options for intervention and treatment. Herein, we utilized commercial *FMR1* TP-PCR reagents to identify the prevalence of FXS in Chinese children with unknown IDD. In addition, the AGG interruption pattern was analyzed in 24 mother-son pairs to investigate the relationship between the AGG structure and characteristics of CGG expansion from mother to child.

## Methods

### Sample recruitment

Patients with unknown moderate to severe IDD (IQ < 55) were recruited from two children hospitals, namely the affiliated Children's Hospital of Capital Institute of Pediatrics and the Peking University First Hospital. The severity of IDD was scored by the Wechsler Intelligence Scale for Children (WISC) [20] or the Gesell Developmental Schedules [21]. DSM-IV criteria were used to indicate a diagnosis of ASD [22]. The recruited patients met at least one of the following requirements:

- Male patient with unknown moderate to severe IDD
- In addition to IDD, the presence of other familial medical problems in the patient’s three-generation pedigree, such as tremor, ataxia, or premature ovarian insufficiency
- Evidence of familial IDD. In addition to the proband, the presence of at least another person with IDD or other neurodevelopmental disorders in the three-generation pedigree, including ASD, developmental delay (e.g., delayed milestones for sitting, walking, or talking), social or behavior problems, learning difficulty or language delay, or ADHD
- Suggested facial dysmorphism, such as a long face, prominent nose and jaw, big ears, thick lip, or other distinctive physical features such as enlarged testicles

Urine screening (GC-MS) was performed on enrolled patients to exclude IDD-related metabolic diseases. Any acquired IDD was also excluded. Of note, this Chinese IDD cohort was previously profiled using aCGH/multiplex ligation-dependent probe amplification (MLPA) and some targeted sequencing [23–26]. Consequently, patients with any IDD-related genomic copy number variants or genomic mutation including subtelomeric aberrations, 16p11.2 microdeletion, 15p11-13 microdeletion or 22q11 microdeletion, or *SHANK3* deletion were excluded. Female patients with Rett Syndrome were also excluded.

Informed consent was obtained from the children's parents in accordance with the publication of any associated clinical information and images. This study was approved by the Capital Institute of Pediatrics and the Peking University First Hospital Review Board.

### DNA extraction and sex identification

DNA was isolated from peripheral blood using the Blood and Tissue kit (Qiagen, Valencia, CA,USA) and quantified with a NanoDrop spectrophotometer (Thermo Scientific, Erembodegem-Aalst, Belgium). The sex of each sample was confirmed after evaluation using a sex-specific PCR assay that targeted the *AMEL* and *SRY* alleles (Additional file 1: Table S1).

### *FMR1* Region-specific CGG PCR

Primers covering the *FMR1* promoter region (Additional file 1: Table S1) were designed to amplify the CGG repeat segment. A modified protocol containing PCR enhancer solution (Life Technology, Grand Island, NY, USA) was used to amplify this GC-rich region. The PCR products were purified (Exonuclease I, New England Biolabs, Ipswich, MA, USA) and sequenced using the standard protocol (BigDye, Applied Biosystems, Foster City, CA, USA). Raw sequences were visualized by Mutation Surveyor V3.30 (SoftGenetics, State College, PA, USA) and blasted to the human reference sequence (http://genome.ucsc.edu/, *hg19*) to determine the CGG repeat number.

### *FMR1* triplet repeat-primed (TP)–PCR

The diluted DNA sample (40 ng/ul) was amplified using AmplideX® *FMR1* PCR reagents (Asuragen, Austin, TX, USA). The PCR product was stored at −20 °C and protected from light before fragment sizing. A 3730xl Genetic Analyzer (Applied Biosystems, Vernon Hills, Illinois, USA) running POP-7 polymer on a 36 cm capillary was used to analyze the amplicon size. A total of 2 ul of unpurified PCR product was mixed with 11 ul Hi-Di Formamide and 2 ul ROX 1000 Size Ladder prior to injection. The raw sequence data was uploaded to GeneMapper 4.0 software (Applied Biosystems, Vernon Hills, Illinois, USA). The size of the PCR product was converted to the repeat number using an MS Excel-based data analysis macro. A mixed internal standard DNA sample was tested in the same plate for each experiment to provide a process control.

Samples were categorized by repeat size as follows. 13–44 CGG repeats were indicated as normal, 45–54 repeats as intermediate, and 55–200 CGG repeats as premutation. Greater than 200 CGG repeats was flagged as full mutation [6]. Six male samples with known CGG repeats (13, 29, 31, 32, 43), and two samples with premutation or full mutation (a gift from Prof. Kun Xia, Central South University, Changsha, China) were assessed as independently genotyped controls. These controls were analyzed by both the *FMR1* region-specific CGG PCR and TP-PCR.

### AGG interruption status, and assessments of maternal transmission to the next generation

Maternal transmissions of CGG repeat alleles were determined in 24 mother-son pairs with different AGG structures. The AGG interruption pattern was deduced from the electropherogram pattern as previously described [16], using both parent and child data to reconcile any ambiguities in interpretation. Any difference in the overall CGG repeat (i.e., >0 repeat) between mother and son indicated CGG expansion following maternal transmission.

## Results

### *FMR1* Region-specific CGG PCR and TP–PCR produce concordant CGG sizing results that are in agreement with known genotypes

**Fig. 1:**
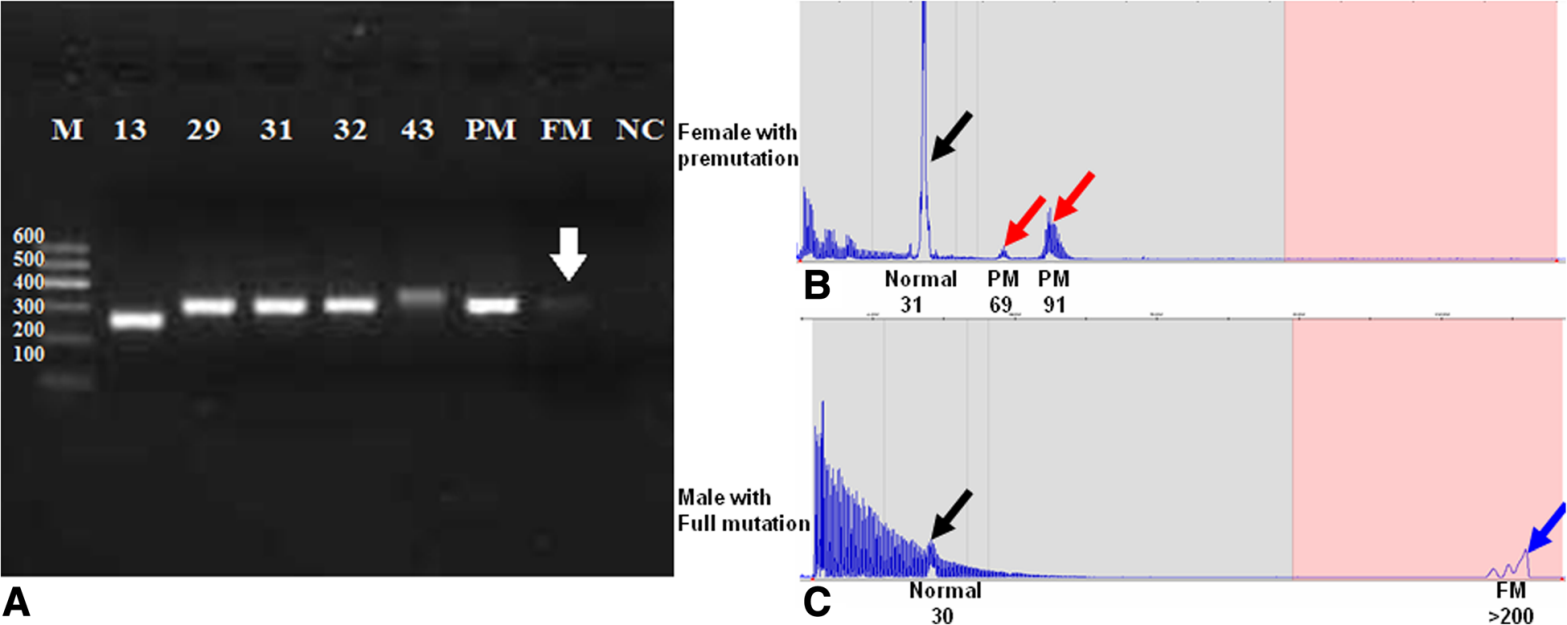
*FMR1* Region-specific CGG PCR and TP-PCR for seven samples with known CGG repeat lengths. **a** The CGG repeat size of seven samples with known genotype were analyzed on agarose gel after *FMR1* region-specific CGG PCR. CGG sizes as determined by the TP-PCR assay or SB analysis is shown at the top of the image. For the premutation (PM) female sample, the gel image reveals the normal *FMR1* allele on another chromosome. For the male sample with a full mutation (FM), the weak band (white arrow) indicates size mosaicism. M: DNA maker; NC: negative control, no DNA for PCR reaction. PCR amplicons from samples with a premutation (**b**) and full mutation (**c**) were also analyzed by capillary electrophoresis following the *FMR1* TP-PCR assay. TP–PCR confirmed size mosaicism in the PM sample (69 and 91 repeats) and the FM sample (30, >200 repeats). This mosaicism was undetected by SB analysis. The black arrow indicates the normal allele, and red arrow indicates the PM allele. The blue arrow indicates the FM allele. The predicted size of the CGG repeats from TP-PCR is labeled

We performed both the Region-specific CGG PCR and TP–PCR assay for seven samples with known CGG sizes. For samples with CGG repeats in the normal size range (13–43 CGG), single well-defined amplification bands were observed on a 2 % agarose gel (Fig. 1a). The sequencing data from region-specific CGG PCR was compared with the predicted sizing from TP–PCR; a deviation of none or one CGG repeat was observed in each case, indicating quantitative consistency in repeat sizing between the two assays. Although the region-specific CGG PCR failed to amplify the sample with >60 repeats (a female premutation, Fig. 1a), the TP–PCR accurately sized this sample (CGG = 31, 69 and 91, Fig. 1b). For the sample with an *FMR1* full mutation, only a very faint amplicon band was observed from the region-specific CGG PCR (white arrow in Fig. 1a). However, the TP–PCR reported a full mutation as well as indications of size mosaicism (CGG = 30 and >200, Fig. 1b). Of note, the size mosaicism detected by TP-PCR was not evident by SB analysis. This is not surprising given the superior sensitivity of TP-PCR compared to SB analysis [16].

### The yield of *FMR1* full mutation in Chinese children with unknown IDD

Samples from 553 unrelated children (male:female = 5.2:1) with unexplained IDD were analyzed using the *FMR1* TP–PCR assay. Samples from 13 children were excluded from data analysis due to unsatisfactory electropherogram traces, leaving 540 patient samples for genotyping. The ratio of isolated IDD and non-isolated IDD was 2.46 (384:156, Table 1); ASD and learning difficulties were common comorbid phenotypes (28.9 %). The normal CGG range was 13–45 repeats, and 29 and 30 repeats represented the most common alleles (62.6 %), consistent with previous Chinese reports [18, 27–29]. Five full mutations were identified from male cases (one case with familial IDD, four cases with sporadic IDD). As a result, the diagnostic yield of *FMR1* full mutation in unknown IDD children was 0.93 % (5/540).

**Table 1:** Clinical information and *FMR1* genotypes of 540 children with unknown IDD

IDD children	Category	*N* (male/female)	FXS yield
		540 (453/87)	
	Isolated IDD (without neuro-developmental comorbid)	384 (307/77)	1
	Non-isolated IDD *	156(146/10)	4
*FMR1* genotyping	Category	N (male/female)	CGG size
	Samples with normal repeat	534(447/87)	13-45
	Samples with full mutation	5 (5/0)	>200
	Samples with intermediate status	1 (1/0)	53

* accompanied by other neuro-developmental comorbid phenotypes, including ASD, ADHD, learning difficulty and seizure/epilepsy

### FXS size mosaicism and phenotypic heterogeneity

Size mosaicism was detected in 4 of 5 FXS patients (80 %), suggesting that CGG size mosaicism is a common phenomenon in FXS patients. Further, intellectual phenotypes were distinct among the 5 FXS individuals. Two patients showed moderate IDD, and whereas 3 demonstrated mild IDD. Three patients also presented other cognitive impairments (eg, learning difficulty/language delay, social dysfunctions). For the sporadic IDD case, facial features characteristic of FXS were not apparent, but they did have large testes.

**Fig. 2:**
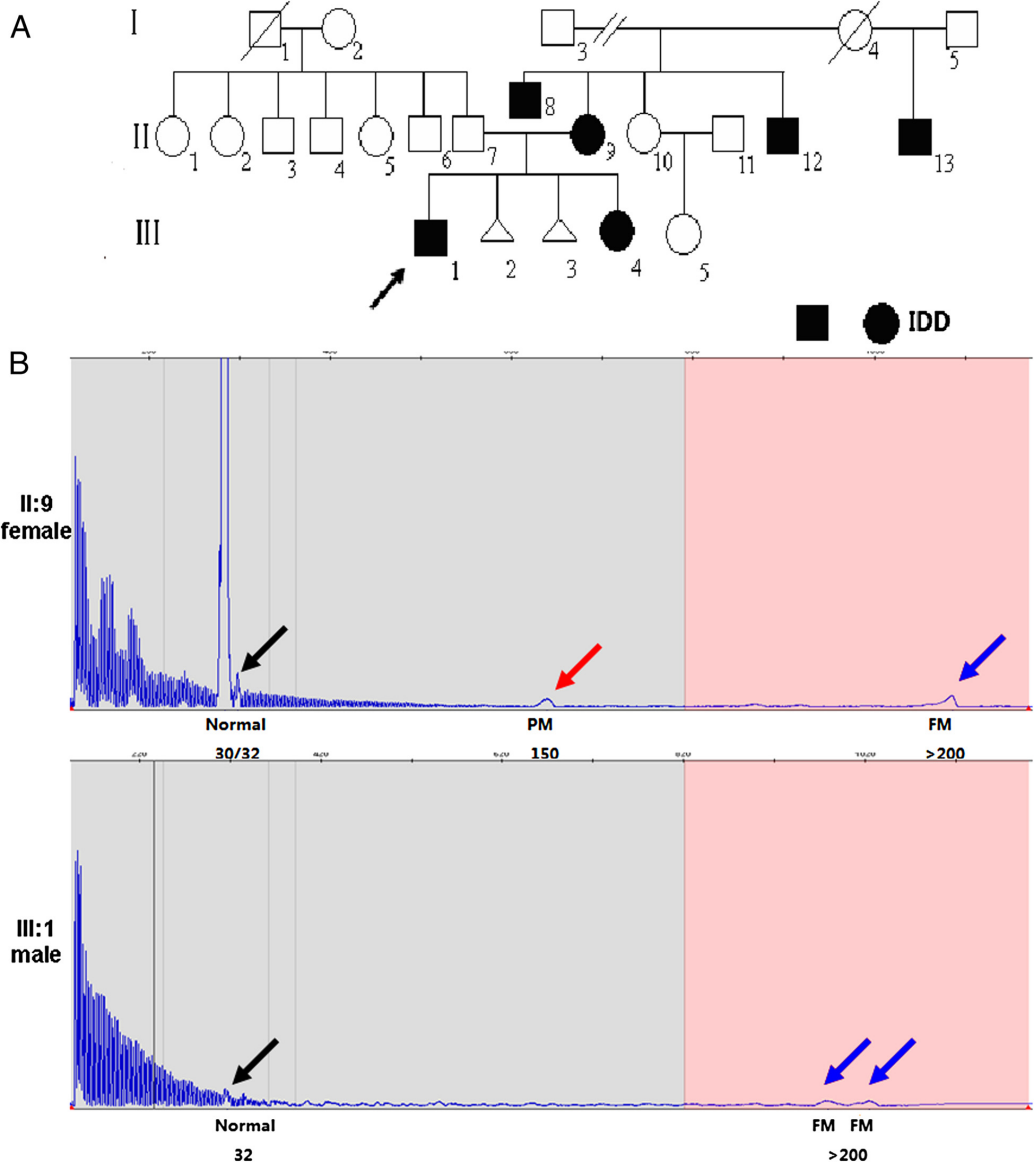
The genotypes and phenotypes for a familial FXS. **a** The pedigree of a classic familial FXS patient (squares indicate males and circles indicate females). IDD-affected individuals are presented as black symbols while normal individuals are presented as white symbols. The proband is indicated with an arrow. The facial features of classic FXS, including long face, predominant nose and large jaw, is seen in the proband (III:1) and his uncle (II:8). **b** Size mosaicism in affected IDD individuals (II:9 and III:1) identified by TP-PCR. The size of CGG repeats is labeled underneath the peak

FXS size mosaicism and phenotypic heterogeneity was particularly prominent in one of the families with a history of IDD (Fig. 2a). The proband in this case study is a sixteen-year-old boy (III:1) with moderate IDD and learning difficulties. His mother (II:9) and younger sister (III:4) showed isolated IDD and his maternal uncles (II:8, II:12) manifested social and cognitive deficits. Of note, one uncle (II:8) presented severe social and cognitive dysfunctions. The predominant facial characteristics of FXS (large nose, big ears and thick lip, and large testes) were seen only in the proband and his uncle (II:8). An *FMR1* full mutation was detected in the proband, his mother, his younger sister and two maternal uncles. Size mosaicism was identified in family members carrying premutation or full mutation.

The maternal expansion of the CGG repeat was further analyzed in II:9 and III:1 (Fig. 2b). The proband’s mother carries one normal *FMR1* allele (CGG = 30, high blue peak), and three mosaic sizes on her affected allele (CGG = 32, black arrow; CGG = 150, red arrow; full mutation, blue arrow). Meanwhile, the proband presented at least two mosaic sizes (CGG = 32, and full mutation). We surmise that the full mutation in the proband was inherited and expanded from the maternal premutation. Alternatively, the mother’s full mutation may be inherited and continued to expand. Any assessment of further expansion, however, would require slab gel analysis of the PCR products using a 2-primer configuration of the AmplideX *FMR1* PCR reagents since these full-mutation amplicons are too large for resolution by CE using the POP-7 polymer.

### Maternal transmissions of CGG repeat in sample with reduced AGG interruption

AGG sequence interruptions in the CGG repeat segment of the *FMR1* promotor region are known to reduce the risk of repeat expansion, possibly by stabilizing strand slippage during DNA replication [30]. For this IDD child cohort, only 21 non-FXS patients had less than two AGG interruptions (21/540 = 3.9 %). This value is similar to previous reports in Chinese populations [29, 31]. Following clinical review and maternal consent, 24 mother-son DNA pairs were available to explore the relationship between AGG interruption and maternal CGG expansion. We subgrouped these 24 samples by AGG interruption status (ten pairs with AGG = 1, fourteen pairs with AGG = 2; see Table 2). Following statistical analysis, the mean maternal CGG repeat was not significantly different between the two groups (29 vs. 30.2, *p* > 0.05). During the maternal transmission, neither the mean size of CGG expansion (0.20 vs. 0.79, *p* > 0.05) nor the frequency of CGG expansion (2/10 vs. 9/14, *p* > 0.05) was significantly different in two subgroups. Consequently, our results indicate that there is no difference between 1 and 2 AGGs with respect to repeat length in the next generation for mothers with normal *FMR1* CGG sizes. This result is consistent with previous findings [32].

**Table 2:** Changes in repeat size during the maternal transmission in 24 mother-son pairs with different AGG structures

Maternal AGG interruption	Number of mother-boy pair	Mean of CGG size in mother*	Mean of CGG expansion during maternal transmission**	Number of mother-boy pair showing CGG expansion (%)
1	10	29	0.20	2 (20 %)
2	14	30.2	0.79	9 (64 %)

* The minimum CGG repeat was determined for mothers with heterozygous *FMR1* alleles; ** The maximum CGG expansion was calculated for mothers with heterozygous *FMR1* alleles; * The difference in CGG size between mother and son CGG genotypes. See Supplementary Table 2 for detailed *FMR1* genotyping information

## Discussion

### *FMR1* TP-PCR is a rapid and accurate method to diagnose FXS in IDD children

Historically, SB analysis has been the standard approach in many clinical laboratories for the molecular diagnosis of FXS patients. However, this process is both time- and labor-intensive (with a typical turn-around time of 2 weeks), requires a large input of DNA, and offers poor resolution to size CGG repeat [16, 33]. Furthermore, SB has a relatively poor analytical sensitivity to detect expansions in samples with low-level size mosaicism [34]. For *FMR1* region-specific CGG PCR, the high GC content of the 5′ untranslated segment is refractory to standard PCR amplification, and usually only <50-100 repeats can be reliably amplified and detected. This inability to reliably identify mid- to large-size expanded alleles can produce false negatives, particularly for female samples where the more likely interpretation of a single amplicon product after electrophoresis is that the *FMR1* allele is homozygous. A further complication is that full-mutation size mosaicism, seen in 4 of 5 FXS patients in our study, can present an additional product peak after PCR that can confound interpretation. Consequently, a more convenient, sensitive, high resolution and accurate assay with a shorter turn-around time is needed to improve the molecular diagnosis of patients that are suspicious for FXS or premutation phenotypes.

The sensitivity and uniformity of the *FMR1* TP-PCR has been previously reported [35]. Our study provides further proof that this single-tube PCR amplification can accurately size CGG repeats from low-input DNA. The total turn-around time from DNA dilution to report acquisition was 48 h, which is considerably less than SB analysis. Moreover, the TP–PCR assay is sensitive enough to detect low-level size mosaicism. We conclude that this *FMR1* TP-PCR is appropriate for FXS molecular diagnosis.

### The prevalence of FXS in Chinese children with unknown IDD

The prevalence of FXS in IDD populations is diverse because both the test method and recruited target populations are diverse. Recently, Peprah reviewed approximately 45 publications that addressed the FXS prevalence in IDD populations [15]. The results revealed a 0.5-9.7 % diagnostic yield of FXS, with Canadian, Estonian, Japanese, and Taiwanese groups having the lowest prevalence of FXS. In addition, countries that don’t routinely perform *FMR1* molecular testing appear to have a significantly lower prevalence than western countries that do [15]. In this study, we determined that the prevalence of FXS in Chinese IDD children is 0.93 % overall, and 1.1 % in male patients and 0 % in female patients. This FXS prevalence is lower than that reported from studies in western counties. A study of 119,232 samples in one large US reference laboratory revealed that the rate of *FMR1* full mutation was 1.3 %, with 1.4 % for males and 0.61 % for females [36]. Another study comprised of 1755 children with non-specific mental retardation reported that the overall yield of FXS was about 3.5 % in a Greek MR cohort [37]. Our study confirmed ethnic differences in FXS prevalence.

We identified a high prevalence of size mosaicism in Chinese FXS boys (80 %). Size mosaicism was reported to be common in FXS patient [38–40]. Such mosaicism can arise *de novo* or be passed on by phenotypically-normal mosaic parents [41, 42]. Recently, an *FMR1* mosaic deletion was reported in a Chinese FXS boy, which was initially absent in his phenotypically-normal mother’s blood. However, an in-depth study of his mother’s *FMR1* profile using qPCR and breakpoint mapping-PCR confirmed low-level mosaicism in different maternal tissues (eg, blood, skin, eyebrow, urine sediment and menstrual discharge) [40]. The maternal inheritance pattern of *FMR1* size mosaicism was also analyzed in the familial FXS pedigree. Evidence of size mosaicism in the proband, his mother, his sister and two uncles suggested that his *FMR1* size mosaicism was inherited from his grandfather.

### Factors affecting the FXS prevalence in Chinese patient populations

The FXS yield from the patients in this study is also different from that reported in previous Chinese IDD/MR populations [18, 19]. Zhong et al. performed multi-institutional screening of 1127 adult/child individuals with mild-moderate IDD, and found that 2.8 % of the Chinese IDD patients carry a full mutation. In this work, both PCR and SB was performed to exclude full mutation and abnormal methylation [18]. In contrast, only a 0.6 % FXS yield was reported from 324 Hong Kong patients with mild mental retardation [19]. For this cohort, electrophoresis of the fragment from the region-specific CGG PCR followed by hybridization using a CGG probe was analyzed to screen FXS. We suspect that both the screening method used and the severity of IDD can affect the determination of FXS prevalence. Further, previous studies have confirmed that FXS appears to be more prevalent among patients with mild MR than severe MR [15, 43]. In this study, severe and moderate IDD accounted for 50 % of recruited patients, and all FXS patients presented moderate IDD.

Both clinical knowledge and complex comorbidities for FXS can also affect the assessment of FXS yield. In China, it is the pediatric neurologist or physician, rather than the clinical geneticist, that examines patients referred for a suspected genetic disorder. However, most Chinese pediatricians, with the exception of some pediatric specialists, are uninformed about FXS. FXS presents a challenging clinical diagnosis based on the fact that the classic facial features of FXS are ambiguous until juvenile development, and that the cognitive presentation of FXS can be subtle in young children [11]. Recently, Li et al. performed a study surveying the FXS knowledge and attitudes of Chinese medical college students [44]. He found that less than one-third of the participants were aware of FXS. This investigation highlights both the challenges and opportunities of genetic education in China. Additionally, many neuro-developmental disorders can occur as major symptoms or comorbid phenotypes in FXS patients. Since a detailed score checklist for FXS testing from GeneReviewers (http://www.ncbi.nlm.nih.gov/books/NBK1384/) was not provided, it would be difficult for pediatrician to distinguish FXS from other possible neuro-developmental disorders. Also, other genomic common copy number variants or rare single nucleotide variants can contribute to variable neuro-developmental traits in FXS [7, 45]. In the future, further background on FXS should be provided to the clinicians. These actions may increase the yield of FXS, perhaps up to 4 % as recently reported [46].

### The role of AGG interruption on maternal *FMR1* CGG expansion

Generally, the normal CGG repeat harbors 2 or 3 AGGs, whereas premutations and full mutations present 0 or 1 AGG [47]. The number of AGGs in the 5’ region was correlated with repeat instability during maternal transmission [32, 47, 48]. Recent studies have demonstrated that women with greater than 54 repeats and no AGG have an elevated risk for expansion to a full mutation in the next generation [32]; However, maternal alleles with <45 repeats rarely expanded, even when they had no or only 1 AGG interruption [32]. In this study, 24 mothers had CGG repeats of <54, and thus our finding that none of these alleles significantly expanded after transmission is consistent with previous reports.

### Limitations of *FMR1* TP-PCR for FXS molecular diagnosis

Over 98 % of FXS is caused by CGG expansions. The remaining cases are attributed to deletions or point variants in the *FMR1* region [49–54]. The ACMG recommends that *FMR1* copy number variants and sequencing of *FMR1* coding regions be performed to exclude possible *FMR1* deletions or point mutations [6] for suspicious cases without repeat expansions. In addition, the abnormal methylation status of the *FMR1* may be associated with FXS [36]. Both deletion/point variants and methylation status of *FMR1* may evade detection by TP-PCR, but methylation analysis can be performed using a separate PCR-based method [34]. Consequently, the above testing is necessary for patients with negative TP-PCR results that are highly suspect for FXS.

## Conclusions

In summary, the *FMR1* TP-PCR assay can accurately and sensitively quantify and classify CGG repeats with a rapid turn-around time. Using this methodology, we established an incidence of 0.93 % FXS in a Chinese children with unknown IDD, and found that size mosaicism was common (80 %) in the 5 patients fragile X full mutations.

## Additional file

Additional file 1:
**Supplementary Table S1.**
