# Supplementary material for: Fragile X syndrome screening in Chinese children with unknown intellectual developmental disorder
Source: BMC Pediatr. 2015 Jul 15;15:77. doi: 10.1186/s12887-015-0394-8 (PMC4502947; doi:10.1186/s12887-015-0394-8)
Supplement: Additional file 1: — Supplementary Table S1. [file 12887_2015_394_MOESM1_ESM.doc]

**Supplementary Table 1 The primes used for sex identification and the *FMR1* CGG region-specific PCR**

| **Name** | **primer sequence** | **Size (bp)** | |
| --- | --- | --- | --- |
|  |  | **chrX** | **chrY** |
| *FMR1-F* | GCTCAGCTCCGTTTCGGTTTCACTTCCGGT | 280 |  |
| *FMR1-R* | AGCCCCGCACTTCCACCACCAGCTCCTCCA |  |  |
| *AMEL-F* | CCCTGGGCTCTGTAAAGAATAGTG | 105 | 111 |
| *AMEL-R* | ATCAGAGCTTAAACTGGGAAGCTG |  |  |
| *SRY-1-F* | CCCGAATTCGACAATGCAATCATATGCTTCTGC |  | 648 |
| *SRY-1-R* | CTGTAGCGGTCCCGTTGCTGCGGTG |  |  |
| *SRY-2-F* | GACAGCAGTAGAGCAGTCAGGGAGG |  | 870 |
| *SRY-2-R* | CTGTAGCGGTCCCGTTGCTGCG |  |  |

**Supplementary Table 2 The detailed *FMR1* CGG sizes and AGG structures for 24 mother-boy pairs**

| **ID of sample** | **CGG size of mother** | **CGG size/AGG count of boy** | **CGG change*** |
| --- | --- | --- | --- |
| 12 | 29/30 | 29/1 | 0 |
| 24 | 29/30 | 30/2 | 0-1 |
| 37 | 29/30 | 29/2 | 0 |
| 82 | 29/30 | 29/2 | 0 |
| 139 | 29/29 | 30/2 | 0-1 |
| 147 | 29/30 | 29/1 | 0 |
| 379 | 29/29 | 29/2 | 0 |
| 395 | 29/29 | 29/2 | 0 |
| 441 | 29/30 | 31/2 | 1-2 |
| 451 | 29/30 | 30/2 | 0-1 |
| 450 | 29/29 | 30/2 | 1 |
| 461 | 29/30 | 31/2 | 1-2 |
| 504 | 29/30 | 30/2 | 0-1 |
| 506 | 29/29 | 30/2 | 1 |
| 25 | 30/31 | 30/1 | 0 |
| 112 | 29/29 | 30/2 | 1 |
| 133 | 29/30 | 29/2 | 0 |
| 1944 | 24/36 | 25/1 | 1 |
| 2559 | 29/29 | 30/1 | 1 |
| 3545 | 33/44 | 33/1 | 0 |
| 4073 | 29/30 | 29/1 | 0 |
| 4565 | 31/36 | 31/1 | 0 |
| 4926 | 29/29 | 29/1 | 0 |
| 5043 | 29/29 | 29/1 | 0 |

* The difference of CGG size between mother and boy represents the index of CGG change.
